# Like Stepping Outside the Noise: Narrative Experiences of Esketamine-Induced Dissociation in Patients with Treatment-Resistant Depression

**DOI:** 10.1192/j.eurpsy.2026.10616

**Published:** 2026-07-31

**Authors:** G. Versaci, M. Olivola, K. La Monica, M. C. Angeletti, V. Casati, A. Frediani, T. Prodi, F. Mazzoni, G. Miacca, A. Guffanti, N. Brondino, V. Martiadis, R. Anniverno, B. M. Dell’osso

**Affiliations:** 1 Mental Health Department, ASST Fatebenefratelli-Sacco; 2 Biomedical and Clinical Sciences Department, University of Milan; 3 Ospedale Macedonio Melloni, ASST Fatebenefratelli-Sacco; 4 Mental Health Department, University of Milan, Milan; 5 Mental Health Department, ASST Pavia, Pavia, Italy; 6 Department of Psychiatry and Behavioral Sciences, Stanford University, Stanford, California, United States; 7 Aldo Ravelli Center for Nanotechnology and Neurostimulation, University of Milan, Milan, Italy

## Abstract

**Introduction:**

Esketamine-induced dissociation is a transient, pharmacologically mediated alteration in consciousness, often distinct from pathological dissociation. While not required for clinical improvement, these states may engage neural circuits implicated in therapeutic change.

**Objectives:**

This study explores patients’ lived experiences of dissociation as potentially meaningful transitional states rather than adverse effects.

**Methods:**

We conducted a qualitative, narrative-based study on 36 patients with treatment-resistant depression (TRD) treated with intranasal esketamine in Northern Italy (2022–2024). Patients provided detailed accounts of dissociative episodes during treatment. Narratives were anonymized and thematically analyzed, focusing on phenomenological dimensions: sensory alterations, temporal distortion, body–space reconfiguration, and emotional detachment from suffering.

**Results:**

Four thematic domains emerged:

1. **Sensory Intensification:** Enhanced colors, synesthetic experiences, and altered auditory landscapes.

2. **Temporal Distortion:** Time dilation, loops, and present-centered awareness.

3. **Body–Space Alteration:** Weightlessness, out-of-body perspectives, and merging with surroundings.

4. **Psychic Distance from Suffering:** Temporary suspension of depressive thoughts and emotional pain.

Most participants described neutral-to-positive experiences, interpreting dissociation as peaceful, transformative, or offering a “pause” from suffering. A minority reported transient fear or disorientation, often mitigated by anticipatory guidance and post-session integration.

**Image 1:**
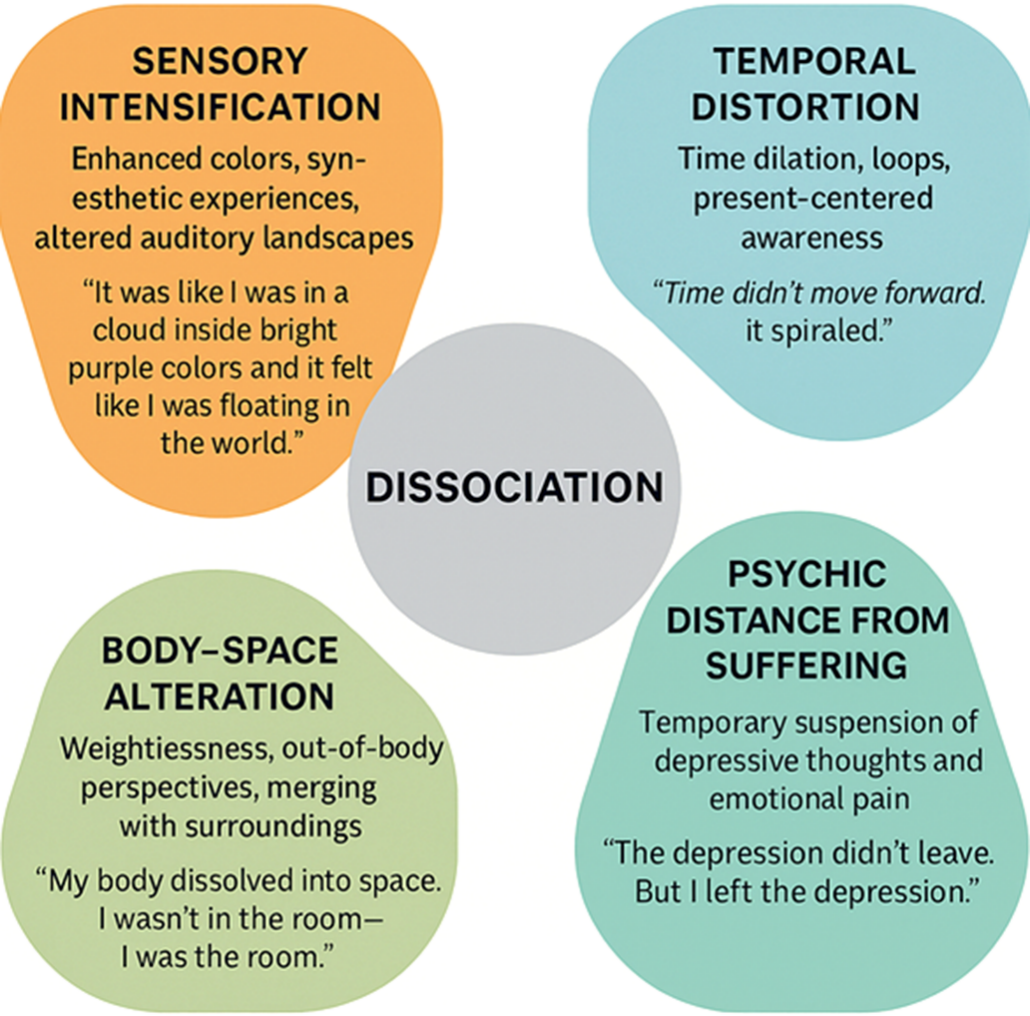
Image 1: Long description.

**Conclusions:**

In TRD patients, esketamine-induced dissociation can function as a liminal mental state that temporarily interrupts maladaptive self-processing. When framed within a supportive therapeutic context, these states may foster relief, insight, and psychological reorganization. Clinical protocols should incorporate psychoeducation and post-treatment reflection to optimize meaning-making and reduce distress.

**Disclosure of Interest:**

None Declared

## Long descriptions

### Image 1: Long description

At the center of the diagram is a grey circle labeled DISSOCIATION. Surrounding this core are four distinct colored organic shapes, each containing a title, a description, and a quote.

* Top-left (Orange): SENSORY INTENSIFICATION. Description: Enhanced colors, synesthetic experiences, altered auditory landscapes. Quote: It was like I was in a cloud inside bright purple colors and it felt like I was floating in the world.

* Top-right (Blue): TEMPORAL DISTORTION. Description: Time dilation, loops, present-centered awareness. Quote: Time didn't move forward. it spiraled.

* Bottom-right (Teal): PSYCHIC DISTANCE FROM SUFFERING. Description: Temporary suspension of depressive thoughts and emotional pain. Quote: The depression didn't leave. But I left the depression.

* Bottom-left (Green): BODY-SPACE ALTERATION. Description: Weightlessness, out-of-body perspectives, merging with surroundings. Quote: My body dissolved into space. I wasn't in the room—I was the room.

Navigate back to Image 1:.

